# Human Digital Twin (HDT) Driven Human-Cyber-Physical Systems: Key Technologies and Applications

**DOI:** 10.1186/s10033-022-00680-w

**Published:** 2022-02-16

**Authors:** Baicun Wang, Huiying Zhou, Geng Yang, Xingyu Li, Huayong Yang

**Affiliations:** 1 State Key Laboratory of Fluid Power and Mechatronic Systems, School of Mechanical Engineering, Zhejiang University, Hangzhou, 310027 China; 2 Department of Mechanical Engineering, University of Michigan, Ann Arbor, MI 48109 USA

## Introduction

Traditional industrial systems, where physical systems, i.e., robots and machines, replaced manual labor mostly, are often known as human-physical system (HPS) [1]. Cyber system, a major outcome of the digitalization revolution, is increasingly adopted in HPS and continuously evolving with the development of advanced manufacturing technologies, leading to the human-cyber-physical system (HCPS) [2]. HCPS fuses human, social networks, physical processes, and cybernetics as an integrated system, characterized by intelligence interaction, diversified integration, and grand system. HCPS connects human, virtual and physical worlds by incorporating intrinsic and abstractive knowledge learned from a variety of manufacturing activities. With the development of advanced sensing, actuation, embedded computing, and artificial intelligence (AI), HCPS will not only create a harmonious human-machine-intelligence collaboration paradigm but also become applicable in a wider range and more sophisticated industrial and health care use cases.

The growing advanced manufacturing technologies have considerably morphed the role and the responsibilities of human in industrial system. In the traditional cyber-physical system (CPS), most research focuses on developing more autonomy, creativity and responsibility of intelligent machines, which inadvertently overlook  human’s importance in critical decision making. One of the major objectives in HCPS is to account for the human in the loop, i.e., shifting from technology-driven approach to a human-centric approach. There are many challenges to realize the HCPS in practice. For example, multi-source context perceptions in the dynamic environment and real-time control of heterogeneous equipment limit the situation awareness of machines thus hindering implementation of HCPS. To tackle the above challenges, the human digital twin (HDT) is introduced to perceive the current context of HCPS and recommend solutions for the human and physical systems.

HDT highlights the importance of human in integrating the physical world and virtual world in HCPS [3]. HDT includes physical representations and virtual models of humans to accurately track and reflect the human motion, perception and manipulation activities and capabilities and to address the challenges in the human-centric manufacturing. Its interconnection and integration among each element regulate human-machine alignment based on human’s proactivity, depict the human-machine interaction and operation performance with the physical and digital representations.

HCPS envisages a system that couples human and physical system to a virtual world. In the context of the HCPS, digitalization of human and physical system helps human to focus on high-value-added tasks and use machines and robots to amplify their impacts on production. Furthermore, increasingly intelligent physical system can free up human capitals and creativity, which makes humans easier to achieve their goal. Here, we propose a HDT-driven HCPS to address such needs to enable human and machines to evolve together in a harmonized way. Moreover, in some special areas, such as the underwater maintenance and space exploration scenarios, the tasks occur in unreachable locations at which, due to accessibility, flexibility, and other technical limitations, the physical representations and virtual models of human are of necessity [2]. To our best knowledge, no referenced HCPS model is structured to appeal to human-centric demands, handle the unstructured working environment and grasp the emerging opportunity that has arisen from the breakthrough in advanced manufacturing technologies. Though studies have been reported the potential application scenarios of HDT in different systems, a unified understanding and summarization of HDT-driven HCPS’s framework and development methods remains to be defined and clarified. The contribution of this paper is to a comprehensive framework of HDT-driven HCPS to address the following issues (1) - distributing skill-based tasks and workload between human and physical system accounting for the variability in manufacturing systems  (2) realizing human-physical system interaction at the system level, providing and enhancing transparency in human and physical systems, and enhancing HCPS’s intelligence regarding analytical assessment, predictive diagnosis, and performance optimization. (3) interconnecting human and the physical system closely and thoroughly by human-machine consistency, and, determining the appropriate level of human-physical system collaboration. In this study, we have discussed the framework, enabling technologies, and application examples of HDT-driven HCPS to fulfill the research gap.

## Framework of HDT-Driven HCPS

**Figure 1:**
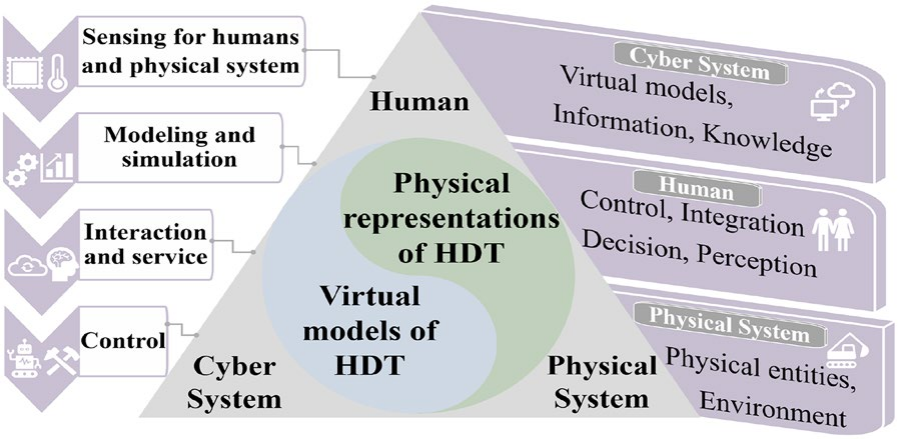
Framework for HDT-driven HCPS

Figure 1 shows the three pillars in the proposed HDT-driven HCPS framework, including human, the physical system, and the cyber system. HDT has evolved into a broader concept that refers to physical representations and virtual models involving HCPS’s elements, which continuously updates and changes as the human change.

As orchestrators of the HDT-driven HCPS, human is at the center of the whole framework and lead the perception, decision, interaction, and control. Data, including decisions and signals, flow between physical system and cyber system, human and physical system, human and cyber system, in real-time. Physical representations of HDT are integrated into the physical system which is the combination of physical entities (i.e., intelligent sensors and actuators) and environments. The physical system assists humans and interacts with humans and the cyber system by receiving teleoperation, thus, HDT-driven HCPS struggles to endow the physical system with more intelligent and more human in their motions. The cyber system reflects the virtual models of HDT, i.e., human’s virtual model and the physical system’s virtual model. Information of the physical system and human is transmitted to the cyber system where information exchange, optimization, and decision-making happen. The cyber system not only takes in human’s knowledge for decision-making but also, in turn, supports knowledge learning, representation and organization of human. In particular, the cyber system not only serves to monitor human and the physical system but also supports the critical decision makings using optimization and AI for continuous improvement of HDT-driven HCPS.

Compared to other proposed HCPS frameworks [1, 4], HDT-driven HCPS moves beyond the state-of-the-art in two main directions: (1) The physical system is human’s physical representation, which will evolve together with human and update the knowledge during human-machine interaction. The development and existence of physical representations of HDT create a balance between the flexibility, adaptability, and efficiency of human and automated assets (e.g., machines, robots). (2) The cyber system manages virtual models of HDT simultaneously, i.e., human’s virtual model and the physical system’s virtual model. It needs to comply with and expand the responsibilities of human in interaction, from analysis to implementation, operation and prediction.

## Enabling Technologies for HDT-Driven HCPS

Enabling technologies for HDT-driven HCPS include Internet of Things (IoT), sensing, AI, cloud computing, robotics. Table 1 summarizes a typical references map of enabling technologies and their implementations in HDT-driven HCPS in the aspects of sensing, computing and analysis, and control.

**Table 1:** Illustration of implementations supported by typical enabling technologies

Enabling technologies	Implementation			
	Sensing	Modeling and simulation	Interaction and service	Control
Flexible sensing	[7]	[8]	[8]	[8]
Motion capture	[5]	[6]	[6]	[18]
Computing and communication	[10]	[9]	[10, 11]	[17]
Artificial intelligence	[12]	[12]	[13]	[13]
Motion mapping and control	[5]	[18]	[5]	[16, 18]
Others related	[12]	[14]	[14]	[19, 20]

### Sensing

Sensing is the cornerstone of HDT-driven HCPS relying on collecting data from human and physical system. Ubiquitous data acquisition supports modeling and quantitative analysis of the whole framework. Related emerging technologies enable and enhance human’s or physical system’s perception and environmental cognition.

#### Sensing for Humans

Sensing devices or smart sensors, which are unobtrusive and miniaturized, acquire not only human motion but also physiological and psychological data, to meet the requirements of multiple HCPS applications. Inertial measurement units [5] and optical technology [6] are applied to capture human motion. Motion data can support virtual model’s formation, and control and interact with physical system. Biological signals, such as electrocardiogram (ECG) and body temperature, are the most commonly used vital tools for monitoring human’s physical condition and diagnosing diseases. By integrating the customized bio-sensing system-on-chip (SoC), the optimized electrodes, and the other circuits, the miniaturized Bio-Patch prototype for single-channel ECG measurement is implemented [7]. It can be foreseen that HDT built upon advanced sensing technologies can directly reflect human’s physiological state or even mental state to model a human more accurately.

#### Sensing for Physical System

To support cooperative dynamic work and realize cognition sharing between the physical world and the virtual world, easy-to-use sensors are responsible for collecting all data from physical system and its encompassed interaction with human and the ambient environment. It aims to connect the physical world and the virtual world by completely capturing the properties and statuses of the physical assets. Multifarious embedded sensors endow robots with perception ability on monitoring key parameters, such as torque, velocity. A soft tactile sensor with self-decoupling and super-resolution abilities achieves super-resolved accuracy [8]. It provides new opportunities in human-robot interaction, such as adaptive grasping, dexterous manipulation [5, 6]. Sensors can be also attached to physical system for ambient environmental cognition, such as humidity, gas concentration. Any sensing components which generate or collect data from physical system and its encompassed assets send the data to the ingestion programs of the cyber system.

### Computing and Analysis

Data computing and analysis enhance the application of the data. It can improve modeling and simulation for the proposed HDT-driven HCPS to outline the virtual world. Advanced applications of data, i.e., interaction and service, can further analyze, evaluate and estimate human and physical system.

#### Modeling and Simulation

**Figure 2:**
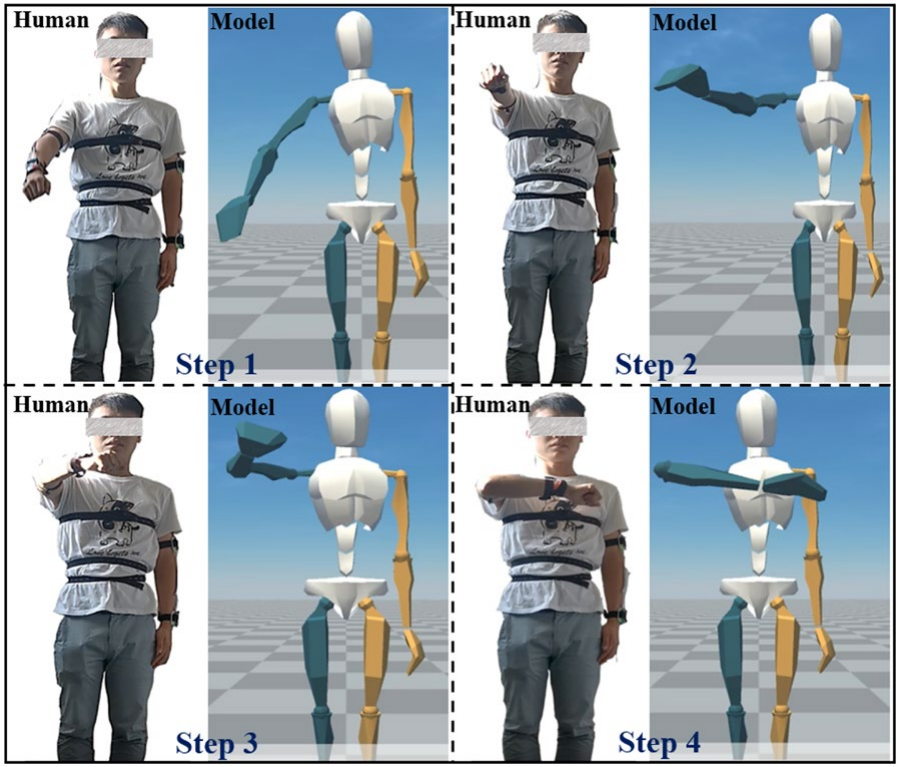
Modeling and simulation of humans

Modeling and simulation (MS) are served as stepping stones in developing virtual world and HDT. In particular, MS of humans is to define and extract key characteristics of humans to create virtual models, as shown in Figure 2. The integrated virtual models mirror the key characteristics and dynamic motion of physical entities and human. Simulation is expected to further explore system performance based on a variety of modeling techniques. Common tools in industrial cases, for example, computer-aided design (CAD) and finite element method simulations, are also used for the HDT-driven HCPS. For example, in an assembly case study, the pre-defined virtual model of robot has been designed in CAD and the digital human model has been represented by deformable mesh [6]. In particular, HDT-driven HCPS emphasizes the system’s dynamic performance which needs bidirectional interactions between the physical system and the cyber system.

#### Interaction and Service

Sub-modules and ambient environments need to interact and serve with each other in HDT-driven HCPS. Interaction and service allocate resources over the whole lifecycle emphatically including design, development, commission, operation, and maintenance. Resources are profiled and applied using related enabling technologies.

5G technology improves signal technology, spectral and signaling efficiency for facilitating data computing and resource allocation. A communication network framework based on 5G technology for cyber-physical IoT systems supports multiple sensors, multiple actuators, and a central controller with the ability of full-duplex communication [9]. The cloud platform provides access to data processing and management in human and physical system. Efforts have been made in developing various applications for human experience, for example, health management, assessing ergonomics performances [10]. The related enabling technologies have penetrated robotic applications gradually. “Robot Cloud” has been brought up to bridge the power of robotics and cloud computing [11]. AI is a branch of computer science that mimics human intelligence to create machine intelligence without reprogramming. Wearable devices with the capability of collecting human physiological signals have been adopted with AI algorithms to classify human actions [12]. Recent advances in AI and deep learning models can guide robots to achieve good performance in motion planning [13]. AI employing little craftsmanship enables the natural evolution for human-robot intelligence in both review-based learning and interaction-based learning paradigm [12].

As the expanding applications put forward higher requirements on interaction and service, more other related technologies are integrated. Extended reality (XR) enhances an interactive and immersive experience of a real-world environment. The XR application using Microsoft HoloLens allows the operator to interact and manage the machine tool and the digital twin simultaneously [14].

### Control

**Figure 3:**
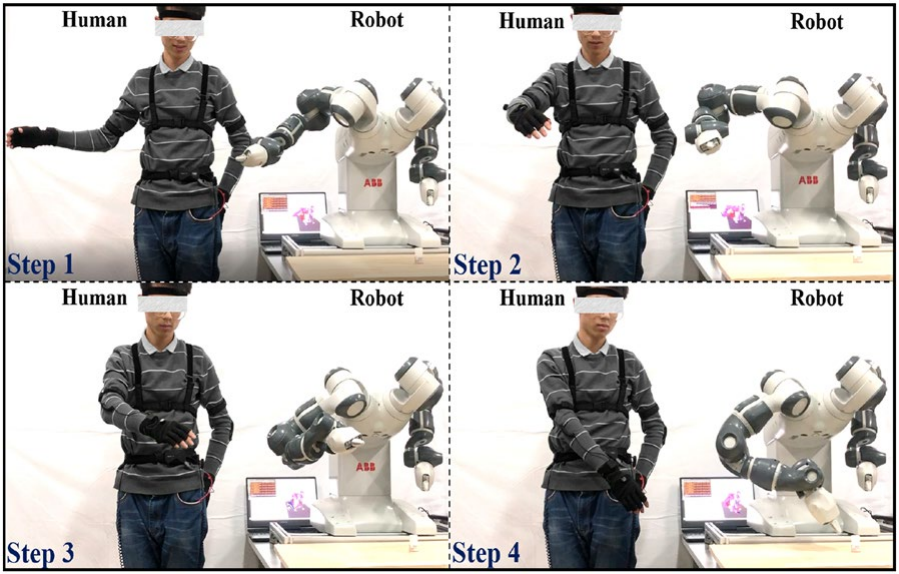
Motion capture and control between human and robot

HDT-driven HCPS allows human to provide commands to and require information from the robot, as shown in Figure 3. Compared to other robotic systems, HDT-driven HCPS’s control frameworks are categorized into three main types described by the style and level of connections between human and physical system, i.e., direct control, shared control, and supervisory control [15]. Direct control represents that machine is controlled by the operator without intelligence or autonomy of itself. Shared control distributes intelligence between the human and the telerobotic system capitalizing on their respective strengths. In supervisory control, the operator makes high-level decisions, and robots autonomously perform tasks with only the state and model information being transmitted to the operator side. The shared control of a dexterous robotic arm with a brain-computer interface and computer vision guidance has been realized. Operators moved the robotic arm to the surrounding area of the target relying on two different mental tasks. With the assistance of the vision guidance, the robotic arm grasped the target autonomously without operators’ accurate command [13]. A reduced-complexity model of the human arm endpoint stiffness for supervisory control has been introduced and evaluated. It provided the operator with the ability to regulate the direction of the major axes of the endpoint stiffness ellipsoid and its volume using natural arm configurations and the contraction of the involved muscles [16].

There exist scientific challenges during designing the teleoperation system’s controller, i.e., modeling for master and slave of teleoperation system, characterizing system uncertainties and disturbances, compensation for communication delay and loss, and satisfying specified performance requirements (e.g., stability, transparency) [17]. Researchers have developed various methodologies to deal with the above challenges in different applications. The mapping relationship between the Denavit-Hartenberg (D-H) models of the human and the robot has been established to map human motions to robots toward intuitive programming [5]. Robot trajectories have been optimized periodically using the data showing the potential of DT to track the interference volumes and the frequency of interferences between human and robots [6].

## Representative Examples

### HDT-Driven HCPS in Isolation Ward

Isolation ward needs a rigorous plan to protect healthcare workers and patients. The proposed HDT-driven HCPS separates human from the physical system for remote operation. Therefore, a teleoperated robot in the isolation ward can not only reduce the risk of infection but also save healthcare workers time for more routines.

A telerobotic system in the isolation ward for Corona Virus Disease 2019 (COVID-19) prevention and control has been developed [18]. The proposed telerobotic system consists of the teleoperation system and the telepresence system. It aims to assist the healthcare workers in the isolation ward. As a human’s second body, it intends to reproduce workers’ professional skills through the robot with lesser training needed. A wearable motion capture suit and a pair of data gloves are used to acquire the healthcare worker’s motion data to control the robot. Teleoperation system has been endowed with diverse functions in view of different healthcare services. The teleoperated system has integrated a dual-arm robot, omnidirectional mobile chassis, and other support devices. It combines human intelligence with robot capabilities to provide services, such as remote auscultation, remote object delivery, and other routine operation. Moreover, the telepresence system deploys a Multi-User Audio/Video Conference System for remote medical consultation and monitoring patient’s emotional states.

### HDT-Driven HCPS in Explosive Ordnance Disposal

Explosive ordnance disposal (EOD) refers to the process to conduct reconnaissance, disposal, and handling of the explosives at the scene of the accident. EOD robot performs the necessary actions whether it is handling EOD or setting related equipment to protect its users from harm’s way. It is integrated with sensors for environmental perception, execution system on the environment, and control system for mapping perception in action. With the development of robotics, researchers have designed and optimized the EOD robot from perspectives of robot control, human-robot interface, and others related [19, 20].

Considering unforeseen changes when robots operated in traditional pre-programmed environments, the new generation concept of EOD robot is designed by the emerging technologies in HDT-driven HCPS. HDT-driven HCPS endows the robot with the ability to perform unstructured tasks, adapt to changes in the environment, and autonomously learn decision-making processes. The EOD robotic system integrates the telerobotic capability into human-robot collaboration to achieve human-in-loop control, which brings great potential in carrying out the EOD tasks. Operator searches and visualizes targets in photo-realistic renderings by employing XR, which blends seamlessly into the natural environment. This effect makes the human accurately control the robot to search and handle explosives giving an unparalleled realism for human. The cyber system allows operators to identify targets by looking at them and retrieving all relevant information from the knowledge repository by leveraging AI and big data.

## Discussions and Conclusions

In this paper, we proposed HDT-driven HCPS to address the human’s needs and repurpose the roles of humans and machines—shifting from the technology-driven approach to a human-centric approach, where humans and the physical systems share own capabilities and intelligence. It features a new perspective on interactions between humans and physical systems, as well as on the related allocation of functionalities and responsibility of each part with the system. Representative enabling technologies are reviewed in terms of sensing, computing and analysis, and control. Furthermore, applications of HDT-driven HCPS as a promising solution in pandemic preventive control and explosive ordnance disposal are discussed. Besides addressing the societal challenges, HDT-driven HCPS is dramatically spilling out to other domains, including, space exploration, marine applications, telemedicine, and disaster response. Typical challenges in HDT-driven HCPS are remarked below:

- Due to the sheer amount of data collection, data exchange, and data processing, HDT-driven HCPS may be hindered by practical issues including safety, privacy, and confidentiality.
- How to characterize interactions, concurrency, and synchronization between continuous cyber systems and discrete physical systems?
- Collaborations and regulations at technical, legal and political levels, are required to advance further development and adaptation of HDT-driven HCPS.
